# Understanding the effect of heat stress during seed filling on nutritional composition and seed yield in chickpea (*Cicer arietinum* L.)

**DOI:** 10.1038/s41598-023-42586-0

**Published:** 2023-09-18

**Authors:** Poonam Devi, Rashmi Awasthi, Uday Jha, Kamal Dev Sharma, P. V. Vara Prasad, Kadambot H. M. Siddique, Manish Roorkiwal, Harsh Nayyar

**Affiliations:** 1 Department of Botany, Panjab University, Chandigarh, India; 2 ICAR-Indian Institute of Pulses Research, Kanpur, India; 3 Department of Agricultural Biotechnology, CSK Himachal Pradesh Agricultural University, Palampur, India; 4 Sustainable Intensification Innovation Lab, Kansas State University, Manhattan, KS USA; 5 The UWA Institute of Agriculture, The University of Western Australia, Perth, WA 6001 Australia; 6 Khalifa Center for Genetic Engineering and Biotechnology, United Arab Emirates University, Al Ain, UAE

**Keywords:** Physiology, Plant sciences

## Abstract

Increasing temperature affects all food crops, thereby reducing their yield potential. Chickpea is a cool-season food legume vital for its nutritive value, but it is sensitive to high temperatures (> 32/20 °C maximum/minimum) during its reproductive and seed-filling stages. This study evaluated the effects of heat stress on yield and qualitative traits of chickpea seeds in a controlled environment. Chickpea genotypes differing in heat sensitivity [two heat-tolerant (HT) and two heat-sensitive (HS)] were raised in pots, initially in an outdoor environment (average 23.5/9.9 °C maximum/minimum), until the beginning of pod set (107–110 days after sowing). At this stage, the plants were moved to a controlled environment in the growth chamber to impose heat stress (32/20 °C) at the seed-filling stage, while maintaining a set of control plants at 25/15 °C. The leaves of heat-stressed plants of the HT and HS genotypes showed considerable membrane damage, altered stomatal conductance, and reduced leaf water content, chlorophyll content, chlorophyll fluorescence, and photosynthetic ability (RuBisCo, sucrose phosphate synthase, and sucrose activities) relative to their corresponding controls. Seed filling duration and seed rate drastically decreased in heat-stressed plants of the HT and HS genotypes, severely reducing seed weight plant^–1^ and single seed weight, especially in the HS genotypes. Yield-related traits, such as pod number, seed number, and harvest index, noticeably decreased in heat-stressed plants and more so in the HS genotypes. Seed components, such as starch, proteins, fats, minerals (Ca, P, and Fe), and storage proteins (albumin, globulins, glutelin, and prolamins), drastically declined, resulting in poor-quality seeds, particularly in the HS genotypes. These findings revealed that heat stress significantly reduced leaf sucrose production, affecting the accumulation of various seed constituents, and leading to poor nutritional quality. The HT genotypes were less affected than the HS genotypes because of the greater stability of their leaf water status and photosynthetic ability, contributing to better yield and seed quality traits in a heat-stressed environment.

## Introduction

Climate change is impacting global food and nutritional security^1^. As a result of the unpredictability of rising temperatures, agricultural yields have dropped sharply as the number of hot days and nights increases while the number of cold days and nights decreases^2–5^. Based on their genetic structure, plants thrive in a preferred temperature range, with exposure above this range causing heat stress (HS)^6^. Changes in the degree and duration of high temperatures affect the entire plant life cycle, including morphological, reproductive, and developmental processes, owing to considerable breakdown of the cellular machinery. Heat stress during the reproductive and seed filling stages limits grain legume production^7^. High temperatures damage membrane thermostability^8^, reduce the relative leaf water content^9^, chlorophyll^10^ and photosynthetic efficiency^11^ to impair cellular functions.

Seed filling, which involves carbohydrate, lipid, and protein synthesis and the mobilization as well as accumulation of various components in developing seeds, is a crucial stage in the life cycle of all leguminous crops^12,13^. Heat stress during seed development affects this process, significantly reducing the seed yield in cereals^14^, legumes^15–19^, and oilseed crops^20,21^. Seed filling rate and seed weight are commonly used to assess the impact of heat stress and tolerance^22^. Reduced seed filling duration decreases seed size in pea, soybean, white lupin^23^, and cowpea^24^. In chickpea^25^ and lentil^7^, high temperatures increase the seed filling rate and decrease seed size. In rice^26^, wheat^27^, and maize^28^, heat stress during seed filling decreases seed size and weight. In addition to yield and yield-related traits, seed quality traits (seed starch accumulation, storage protein, and minerals) are negatively affected by heat stress in various crops, including several grain legumes^3,7,13,29,30^. Heat stress inhibits starch accumulation in seeds of cereals^31^, such as wheat^32^, rice^33^, and maize^34^, which could be a major factor affecting seed size. High temperatures affect protein accumulation in pea^35^ and soybeans^5,36^, lentil^29^, lupin^37^, chickpea^30^, and the oil content in oliferous crops^38^.

Chickpea performs well when the reproductive stage coincides with the optimum temperature (20–28 °C)^39^. As a heat-sensitive crop, chickpea is particularly vulnerable to elevated temperatures during critical stages of its growth and development. Heat stress can substantially affect chickpea plant growth and development. This can adversely influence the seed germination and seedling emergence^40^. Heat stress in the late vegetative stage of chickpea plants causes noticeable symptoms, such as leaf scorching, burning of stems and leaves, leaf rolling, leaf tip damage, leaf drying, and senescence^41,42^. In addition, heat stress during the reproductive stage frequently induces flower abscission or shedding, decreasing in the number of flowers^14,21,41,43^. Heat stress can reduce pollen grain and stigma viability, disrupt fertilization, and impede the growth of developing flowers and pods^25,39,41,43^. Consequently, pod abortion increases, reducing pod formation^37,42,44^. Thus, due to heat stress, chickpea plants produce fewer seeds with lower weight^39,41,42,44^. Heat stress can have a wide range of detrimental effects on various aspects of chickpea plants, including physiological and biochemical traits^25,44^. Chickpea is generally grown as a rain-fed crop during the winter season (November to April) in northern India and often experiences high-temperature (> 32 °C) stress during the reproductive and seed-filling phases (mid-February to April), especially in a late-sown environment^40^, adversely affecting seed development^30^.

Thus, the present study was specifically designed to investigate the effects of heat stress at the time of seed development on seed-filling processes and the consequent effects on nutritional quality in chickpeas, which have not been reported previously. This study involved contrasting genotypes for heat sensitivity, as identified in our previous study, to identify the target sites of heat stress and the probable mechanisms of heat tolerance. It was hypothesised that the heat-tolerant genotypes of chickpea would have less impact on seed development characteristics, including seed weight, seed size, and seed yield, compared to the heat-sensitive genotypes when exposed to heat stress.

## Materials and methods

### Raising plants

**Figure 1:**
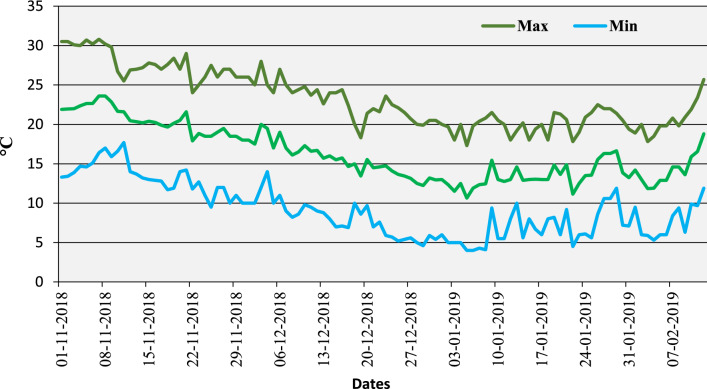
Temperature profile (Max., Min., Average) for plants grown in outdoor environment average temperature as 23.5/9.9 °C; day/night) prior to exposing them to heat stress under controlled environment (32/20 °C).

The seeds of four chickpea genotypes, two heat-tolerant (ICCV07110 and ICCV92944) and two heat-sensitive (ICC14183 and ICC5912), procured from the International Crops Research Institute for the Semi-Arid Tropics (ICRISAT), India, were raised in pots (8 kg capacity) filled with a mixture of air-dried soil (available N, P, and K of 54, 43, and 158 kg ha^−1^, respectively; loam; pH 7.1), sand, and farmyard manure (2:1:1 (v/v) ratio). The seeds were treated with a suitable *Mesorhizobium ciceri* strain (2.0 g kg^−1^ seed) before being sown in pots on November 1, 2018, at Panjab University, Chandigarh, India (30°45′38.2″ N,76°45′55.4″ E). Three seeds per pot were planted and thinned to two healthy plants after establishment. The plants were grown in a natural outdoor environment with an average temperature of 23.5/9.9 °C (day/night; Fig. 1), relative humidity of 63.4/39.5%, and light intensity of ~ 1,300–1,510 μmol m^−2^ s^−1^ until the beginning of pod set (107–110 d after sowing). At this stage, the pots were transferred to controlled-environment growth chambers, with half (five pots in three replicates; 5 × 3 = 15 pots per genotype) maintained at 25/15 °C (control) and the other half exposed to 32/20 °C (heat stress) until maturity.

### Leaf function evaluation

#### Membrane damage

Fresh leaves, collected from the top branches of the control and stressed plants 10 days after initiation of pod filling (DAP), were tested for membrane damage using the electrolyte leakage method^45^. The leaves were washed with deionized water to eliminate any surface contaminants, placed in glass vials with deionized water (10 mL), and incubated at 25 °C for 24 h on a rotary shaker. Thereafter, electrical conductivity was measured using a conductivity meter.

#### Relative leaf water content

Leaf water status was measured by recording the fresh, turgid, and dry weights of the same leaves, collected at 10 DAP, according to the method described by Barrs and Weatherley^46^. Leaf stomatal conductance was recorded on the same leaves using a leaf porometer as described previously^47^.

#### Chlorophyll concentration and chlorophyll fluorescence

Chlorophyll (Chl) concentration was determined in fresh leaves collected from the top branches at 10 DAP and extracted in 80% acetone. The extracts were centrifuged at 10,000 rpm, and the absorbance was measured spectrophotometrically at 645 and 663 nm using the method described by Arnon et al.^48^. Photochemical efficiency, indicating PSII function, was measured as Chl fluorescence in the fresh leaves of the top branches at 11:00 using a modulated chlorophyll fluorometer (OS1-FL, Opti-Sciences, Tyngsboro, MA, USA), as detailed elsewhere^25^.

#### RuBisCo activity

RuBisCo activity was assayed in fresh leaves collected from top branches at 10 DAP. The leaves were homogenized in a pre-cooled mortar and pestle with a buffer solution containing 50 mM 1,3-bis tris (hydroxymethyl) methylamino propane (pH = 7.0), 10 mM NaHCO_3_, 10 mM MgCl_2_, 1 mM EDTA,10 mM DTT, 1 mM benzamidine, 1.5% polyvinyl polypyrrolidone, 1 mM phenylmethyl-sulfonyl fluoride, and 3 mM 3-methylbut-2-ene-1-thiol^49^. The extract was centrifuged at 10,000 rpm for 40 min, the supernatant was desalted and assayed according to the method described by Racker^50^and elaborated elsewhere^47^.

#### Sucrose synthase activity

Fresh leaves collected at 10 DAP, were homogenized in an ice-cold extraction medium containing HEPES/KOH buffer (200 mM; pH 7.8), 3 mM magnesium acetate,10 mM dithiothreitol (DTT), and 3 mM EDTANa_2_.2H_2_O. The homogenate was centrifuged at 10,000 rpm for 20 min at 4 °C, and the supernatant was collected for enzyme activity analysis, as per the method described by Xu et al.^51^.

#### Sucrose

Leaf sucrose was measured from fresh leaves extracted in 80% ethanol three times at 80 °C for 1.5 h. The extracts were evaporated at 40 °C in an air-circulating oven. Sucrose was measured using the method described by Jones et al.^52^, as detailed earlier^53^.

#### Seed composition

Starch, sugar, protein, and fat contents were measured in the mature seeds. For the extraction of starch and sugars, seeds were homogenized with 30% (v/v) perchloric acid and 95% (v/v) ethanol, respectively, and their contents were estimated according to the method described by Dubois et al.^54^, using glucose (Sigma D9434; Sigma, WI, USA) as a standard. Crude proteins, crude fats, and minerals were analyzed according to AOAC standard procedures^55^.

Storage proteins were sequentially fractionated using the method described by Triboi et al.^56^. Mature seeds were homogenized in wholemeal flour. At each extraction step, the seed samples were continuously stirred for 60 min using a magnetic stirrer. The samples were centrifuged at 10,000 rpm for 30 min at extraction temperature to separate the soluble and insoluble fractions. Albumins and globulins were extracted with 25 mL sodium phosphate buffer (0.05 M; pH 7.8) and NaCl (0.05 M), respectively, at 4 °C. The prolamins were extracted from the previous pellet at 20 °C using 25 mL of 70% (v/v) ethanol. Similarly, glutelins were extracted from the previous pellet at 20 °C with 25 mL of 20 gL^−1^ sodium dodecyl sulfate (SDS), 2% (v/v) 2-mercaptoethanol (2-SH), and 0.05 M tetraborate buffer (pH 8.5). These were obtained from the supernatant after centrifugation at 10,000 rpm. The protein concentration in each fraction was measured according to the method described by Lowry et al.^57^.

#### Seed carbohydrates

Carbohydrates, such as glucose, fructose, and sucrose, were measured as described by Hu et al.^58^. From the seed extract, 20 µL was placed in an oven at 50 °C for 40 min to evaporate ethanol. Subsequently, 20 µL of distilled water was added to each sample, followed by the addition of 100 μL glucose assay reagent (Sigma), mixed, which were then heated at 30 °C for 15 min. The absorbance was measured at 340 nm to calculate the glucose content. Thereafter, each sample was heated twice with 0.25 U phospho-glucose isomerase at 30 °C for 15 min and then 83 U invertase for 60 min. After each incubation step, the absorbance was measured at 340 nm to estimate fructose and sucrose levels.

#### Seed growth rate and seed-filling duration

To measure seed growth rate, five pods plant^–1^ were tagged at the initiation of pod filling (pod size: ~ 1 cm). Seed dry weight was measured seven days after the onset of pod filling and at physiological maturity. The difference between the seed dry weights at the two stages, divided by the number of days to reach physiological maturity, indicated the seed filling rate day^–1^. The seed-filling duration for tagged pods was calculated by recording the number of days from the onset of seed filling to physiological maturity.

#### Yield traits

Yield traits (seed weight, seed number plant^–1^, and individual seed weight) were recorded from ten plants genotype^–1^ treatment^–1^; replicate data were pooled and presented.

### Statistical analysis

#### The experiment was conducted using randomised block design

Observations were replicated three times, and the data were analysed for means and standard errors. ANOVA was conducted, with the least significant difference (LSD) calculated (*P* < 0.05) using AGRISTAT software.

### Research involving plants

The seeds of four chickpea genotypes, two heat-tolerant (ICCV07110 and ICCV92944) and two heat-sensitive (ICC14183 and ICC5912), were obtained from the International Crops Research Institute for the Semi-Arid Tropics (ICRISAT), gene bank, India following the international norms, legislation and guidelines.

## Results

ANOVA (2-way) Tables 1 and 2 show the mean sum of squares and significance for the genotype × treatment interaction, respectively, for various traits.

**Table 1:** ANOVA values for mean sum of squares in leaf and seed traits across the four chickpea genotypes under heat stress.

Yield and seed traits														
Source of variation	d.f	Total weight plant^−1^	Seed yield plant^−1^	Avg. seed weight	Avg. seed size	Pods plant^−1^	Seed No. 100 pods ^−1^	HI	Seed growth rate	Seed fill duration	Sucrose (Seed)	SS (Seed)	Ssy	AIs
Genotypes	3	21.03**	5.6**	1330.9**	9.2**	38.4**	1759.00	0.023**	7.45**	54.4**	84.4**	120.7**	168,363**	113,775.5**
Replication	2	0.4	0.03	201.3	0.04	2.2	0.11	0.002	0.46	2.88	31.2	17.80	443.80	1190
Error	6	1.7	0.2	1.2	0.2	2.6	15.3	0.001	0.14	2.5	0.01	1.01	171.7	8.5

Seed constituents														
Source of variation	d.f	Albumins	Globulins	Glutelins	Prolamins	Starch	Proteins	Fat	Crude fiber	Ash	Soluble sugars	Glucose	Fructose	
Genotypes	3	16.2**	281.4**	61.7**	4.1**	135.2**	47.4**	1.68**	1.89	0.04	0.84	3.7**	3.7**	
Replication	2	0.6	5.7	4.8	0.4	26.2	4.8	0.01	0.01	0.6	0.5	1.8	2.3	
Error	6	0.2	3.7	61.7	0.3	0.07	2.1	0.4	0.4	0.88	0.41	0.1	0.05	

**Significance at *P* < 0.05; *** at *P* < 0.01.

EL: electrolyte leakage, Chl: chlorophyll, ChlF: Chlorphyll fluorescence, gS: stomatal conductance, SS: sucrose synthase SS: sucrose synthase, Ssy: starch synthase, AI: Acid invertases.

**Table 2:** Analysis of variance (ANOVA) showing statistical significance in various studied traits measured in 4 genotypes under non-stress and heat stress.

Traits	Genotype	Treatment	Interaction
Electrolyte leakage	***	***	***
Chlorophyll	***	***	***
Chlorophyll Fluorescence	***	***	***
Relative leaf water content	***	***	***
Stomatal conductance	***	***	***
RuBisCo	***	***	***
Sucrose synthase (leaf)	***	***	***
Sucrose (leaf)	***	***	***
Total weight plant ^−1^	***	***	***
Seed yield plant^−1^	***	***	***
Average seed weight	***	***	***
Average seed size	***	***	***
Pods plant^−1^	***	***	***
Seed number 100 pods^−1^	***	***	***
Harvest index	***	***	***
Seed growth rate	**	**	**
Seed fill duration	**	**	**
Starch (seed)	**	**	**
Proteins(seed)	**	**	**
Fat(seed)	ns	**	**
Crude fiber (seed)	**	**	ns
Ash (seed)	Ns	**	**
Soluble sugars (seed)	Ns	**	ns
Albumins (seed)	***	***	***
Globulins (seed)	***	***	***
Glutelins (seed)	***	***	***
Prolamins (seed)	**	**	**
Sucrose (seed)	**	**	**
Glucose (seed)	**	**	ns
Fructose (seed)	**	**	ns
Sucrose synthase (seed)	***	***	**
Soluble starch synthase(seed)	***	***	***
Acid invertases(seed)	**	**	***

** Significance at *P* < 0.05, *** significance at *P* < 0.01, ns = non-significant.

### Leaf membrane damage

**Figure 2:**
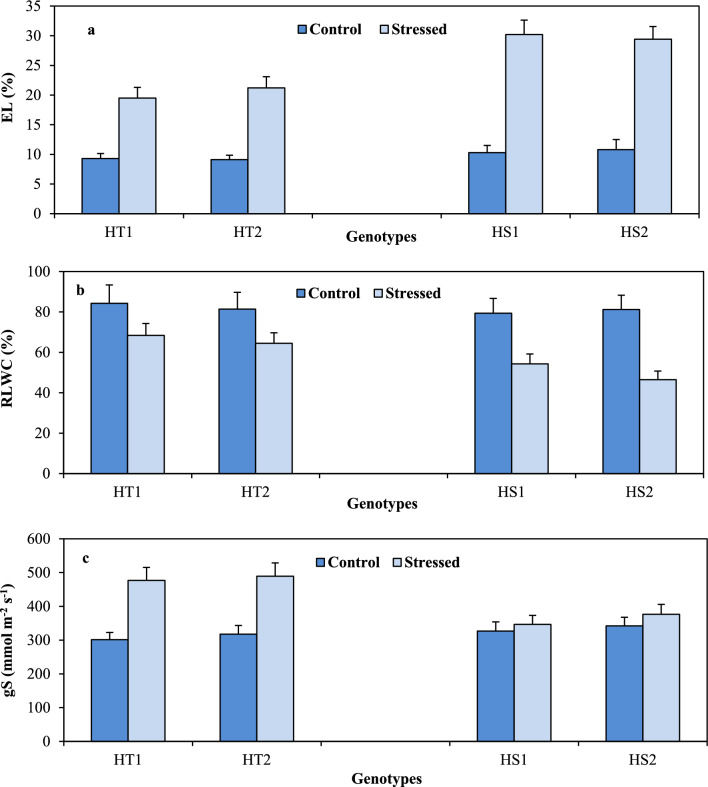
Membrane damage (as electrolyte leakage %; (**a**)), relative leaf water content (RLWC; (**b**)) and stomatal conductance (gS; (**c**)) in heat-tolerant (HT1: ICCV07110; HT2: ICCV92944) and heat-sensitive (HS1: ICC14183; HS2: ICC5912) chickpea genotypes grown under control (25/15 °C) and heat stress (32/20 °C) environment. LSD: (*P* < 0.05) (genotypes x treatment interaction): Chl: 2.65; RLWC: 9.76; gS: 32.8.

Leaf membrane damage (expressed as % electrolyte leakage) in the control plants varied from 9.1 to 9.3% in the HT genotypes and 10.3–10.8% in the HS genotypes (Fig. 2A). Heat stress increased membrane damage more in the HS genotypes (29.4–30.2%) than in the HT genotypes (19.5–21.2%) relative to their corresponding controls (Tables 1, 2).

### Leaf water status

Leaf water status [(as relative leaf water content (RLWC; %)] in the control plants differed from 81.4 to 84.3% in the HT genotypes and 79.4–81.2% in the HS genotypes (Fig. 2B). Heat stress decreased RLWC in the HS genotypes more than that in the HT genotypes compared to their respective controls.

The stomatal conductance (gS) was in the range of 301–317 mmol m^–2^ s^–1^ in the HT genotypes and 326–342 mmol m^–2^ s^–1^ in the HS genotypes (Fig. 2C). Heat stress increased gS much more in the HT genotypes (54–58%) than in the HS genotypes (6–10%) relative to their corresponding controls. (Tables 1, 2).

### Photosynthetic ability

**Figure 3:**
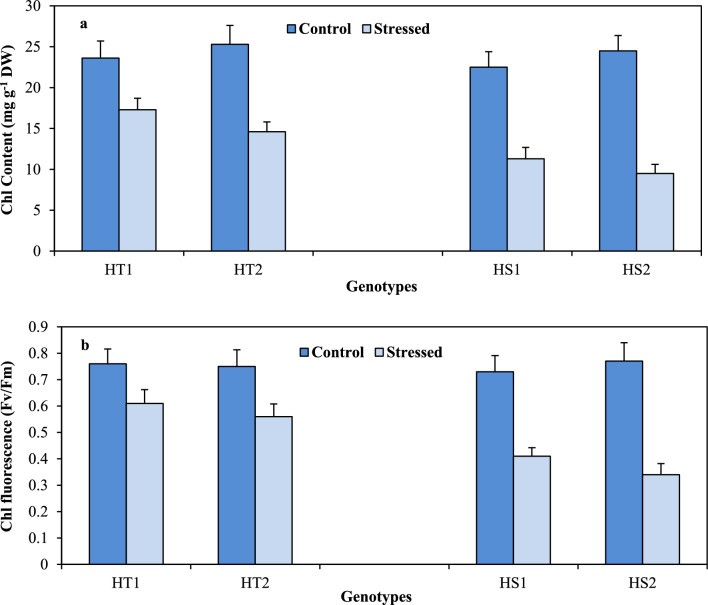
Chlorophyll content (Chl; a) and Chl fluorescence (ChlF; b) in heat-tolerant (HT1: ICCV07110; HT2: ICCV92944) and heat-sensitive (HS1: ICC14183; HS2: ICC5912) chickpea genotypes grown under control (25/15 °C) and heat stress (32/20 °C) environment. LSD: (*P* < 0.05) (genotypes x treatment interaction): Chl: 2.65; Chl F: 0.059.

Leaf Chl concentration (Fig. 3A), an indicator of the stay-green trait, in the control plants varied from 23.6 to 25.3 mg g^–1^ DW in the HT genotypes and 22.5–24.5 mg g^–^^1^DW in the HS genotypes. Under heat stress, Chl concentration decreased more significantly in the HS genotypes (49–61%) than in the HT genotypes (23–26%) relative to their respective control groups (Tables 1, 2).

Photosynthetic efficiency, as indicated by chlorophyll fluorescence (Fig. 3B), was measured as the Fv/Fm ratio. In the control plants, in the HT genotypes, it was in the range of 0.75 to 0.76, while in the HS genotypes, it varied from 0.73 to 0.77. When exposed to heat stress, the Fv/Fm ratio decreased more prominently in the HS genotypes (44–56%) than in the HT genotypes (19.7–25.3%) relative to their respective control groups (Tables 1, 2).

**Figure 4:**
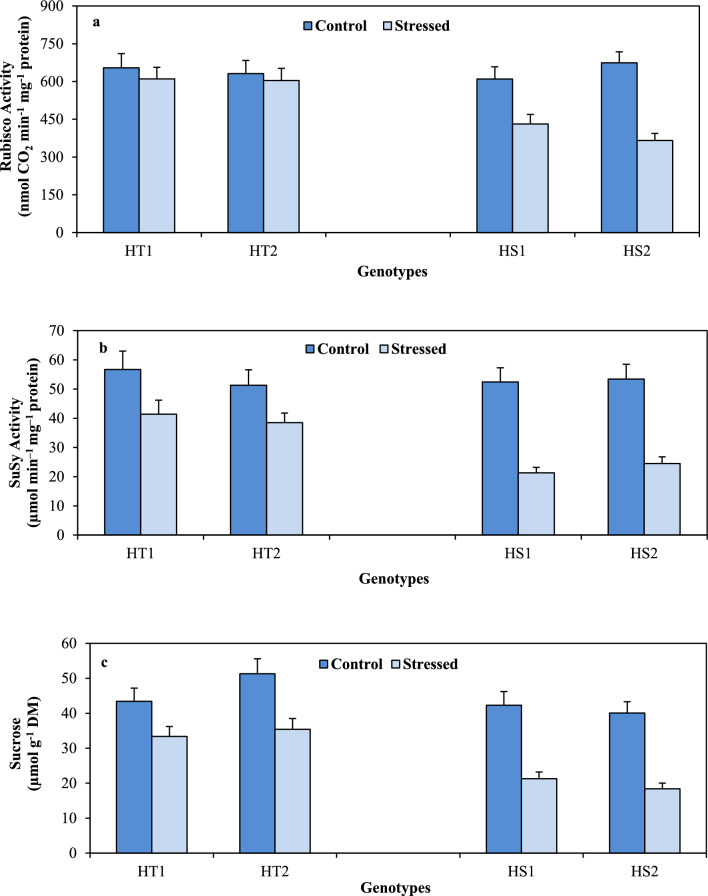
Ribulose 1,5 bisphosphate carboxylase (RuBisCo; a), Sucrose synthase (SS; b) and sucrose (Suc; c) in heat-tolerant (HT1: ICCV07110; HT2: ICCV92944) and heat-sensitive (HS1: ICC14183; HS2: ICC5912) chickpea genotypes grown under control (25/15 °C) and heat stress (32/20 °C) environment. LSD: (*P* < 0.05) (genotypes x treatment interaction): RuBisCo: 36.8; SS: 6.8; Suc: 6.1.

RuBisCo activity (Fig. 4A)—an indicator of carbon fixation ability in control plants, exhibited values between 631 and 654 nmol CO_2_ min^–1^ mg^–1^ protein in HT genotypes and 609–674 nmol CO_2_ min^–1^ mg^–1^ protein in HS genotypes. Heat stress reduced RuBisCo activity more in the HS genotypes (29–46%) than in the HT genotypes (10–12%) relative to their corresponding controls (Tables 1, 2).

Sucrose synthase activity (Fig. 4B) in the control plants differed from 51 to 56 µmol min^–1^ mg^–1^ protein in the HT genotypes and 52–53 µmol min^–1^ mg^–1^ protein in the HS genotypes. Heat stress decreased sucrose synthase activity more in the HS genotypes (54–59%) than in the HT genotypes (24–26.9%) relative to their corresponding controls (Tables 1, 2).

Leaf sucrose concentrations (Fig. 4C) in the control plants varied from 43.4 to 51.3 µmol g^–1^ DW in the HT genotypes and 40.1–42.3 µmol g^–1^ DW in the HS genotypes. Heat stress reduced leaf sucrose concentration more in the HS genotypes (49.6–54.1%) than in the HT genotypes (23–30%) relative to their respective controls (Tables 1, 2).

### Yield and yield-related traits

Yield traits ((Tables 1, 2, 3) included total plant dry weight (aboveground) plant^–1^, seed weight plant^–1^, average seed weight, average seed size, pod number plant^–1^, and seed number per 100 pods.

**Table 3:** Yield traits, seed growth rate, and seed filling rate in heat-tolerant (HT1: ICCV07110; HT2: ICCV92944) and heat-sensitive (HS1: ICC14183; HS2: ICC5912) chickpea genotypes grown in control (25/15 °C) and heat stress (32/20 °C) environments.

Traits	Control				Stressed			
	HT1	HT2	HS1	HS2	HT1	HT2	HS1	HS2
Total dry weight plant^–1^ (TDW)	13.1 ± 1.15a	11.6 ± 1.13a	12.1 ± 1.11a	10.6 ± 1.13a	9.5 ± 1.1b	8.7 ± 0.89b	4.56 ± 0.87c	4.14 ± 0.84c
Seed yield plant^–1^ (SY)	5.7 ± 0.56a	5.1 ± 0.45a	5.1 ± 0.36a	4.8 ± 0.42a	4.1 ± 0.51b	3.7 ± 0.34b	1.98 ± 0.22c	1.34 ± 0.20c
Average seed weight (mg) (SW)	106.7 ± 8.5a	101.5 ± 8.7a	103.5 ± 9.4a	101.9 ± 8.5a	78.5 ± 6.1b	67.9 ± 7.3b	40.1 ± 4.5c	34.6 ± 3.9d
Average seed size (mm) (SS)	6.1 ± 0.61a	5.8 ± 0.58a	5.6 ± 0.48a	5.3 ± 0.41a	4.6 ± 0.45b	4.2 ± 0.39b	2.24 ± 0.43c	2.11 ± 0.18c
Pod number plant^–1^ (PN)	20.4 ± 1.13a	18.3 ± 1.11a	16.7 ± 1.31b	15.4 ± 1.21b	14.3 ± 1.43bc	12.8 ± 1.32c	7.2 ± 1.17d	6.92 ± 1.16d
Seed number 100 pods^–1^ (SN)	103.4 ± 6.2a	98.3 ± 6.1a	91.3 ± 5.9b	87.6 ± 6.5b	78.9 ± 7.1c	71.3 ± 6.8c	35.3 ± 3.7d	32.3 ± 2.9d
Harvest index (HI)	0.43 ± 0.076a	0.41 ± 0.056a	0.39 ± 0.043bc	0.37 ± 0.037c	0.3 ± 0.019d	0.27 ± 0.031d	0.14 ± 0.021e	0.12 ± 0.018e
Seed growth rate (mg seed^–1^ day^–1^) (SGR)	7.6 ± 0.87a	7.3 ± 0.65a	7.1 ± 0.87a	7.2 ± 0.59a	5.7 ± 0.64b	5.1 ± 0.71b	2.9 ± 0.59c	2.67 ± 0.43c
Seed fill duration (days) (SFD)	23.1 ± 1.54a	20.4 ± 1.37a	21.3 ± 1.76a	19.5 ± 1.35bc	16.6 ± 1.54c	15.1 ± 1.78c	8.9 ± 1.55d	8.65 ± 1.39d

**LSD (P** <  < 0.05) for genotypes x treatment interaction: TDW (2.3), SY (1.5), SW (10.2), SS (1.3), PN (2.1), SN (7.6), HI (0.083), SGR (1.13), SFD (2.11).

Values represent mean ± S.E. Different lower-case letters for each trait indicate significant variations (*P* < 0.05).

In relation to their respective control groups, following impacts were observed due to heat stress.

**Figure 5:**
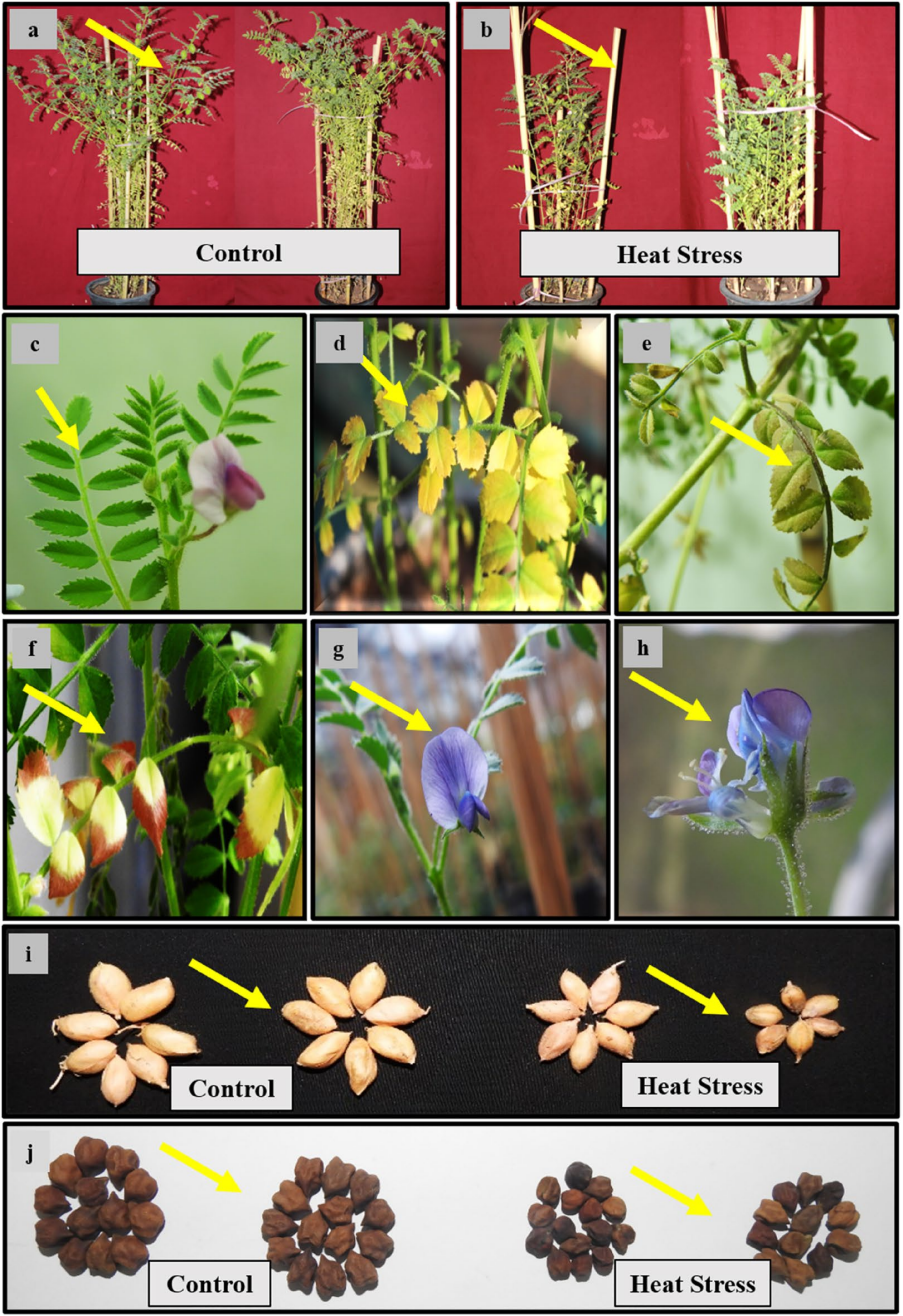
Morphological effects of heat stress (HS) observed on chickpea plants: plant height with more number of pods; under control (**a**), reduced plant height with lower number of pods; under HS (**b**), healthy leaves; under control (**c**), leaf chlorosis under HS (**d**), leaves necrosis; under HS (**e**), leaf scorching/leaf bleaching of leaflets due to photooxidation under HS (**f**), healthy flower; under the control (**g**), aborted flower under HS (**h**), comparative pod size under control and HS (**i**) and comparative seed size under control and HS environment (**j**).

- Total plant dry weight: Heat stress caused a reduction of 60–62% in the HS genotypes and 25–27.4% in the HT genotypes.
- Pod number per plant: Heat stress resulted in a decrease of 55–57% in the HS genotypes and 29–30% in the HT genotypes (see Fig. 5).
- Seed number per 100 pods: Heat stress led to a reduction of 62–63% in the HS genotypes and 23–27% in the HT genotypes (see Fig. 5).
- Average seed weight: Heat stress caused a decrease of 62–66% in the HS genotypes and 26–32% in the HT genotypes (see Fig. 5).
- Average seed size: Heat stress resulted in a decrease of 60% in the HS genotypes and 24–27% in the HT genotypes.
- Seed yield per plant: Heat stress led to a decrease of 61–72% in the HS genotypes and 27–29% in the HT genotypes.
- Harvest index: Heat stress caused a reduction of 64–67% in the HS genotypes and 30–34% in the HT genotypes.

### Seed-filling rate and duration

Heat stress decreased the seed-filling rate by 59–63% and the seed-filling duration by 55–58% in the HS genotypes relative to their corresponding controls, with reductions of 25–31% and 25–28%, respectively, in the HT genotypes (Tables 1, 2, 3).

### Seed components

**Figure 6:**
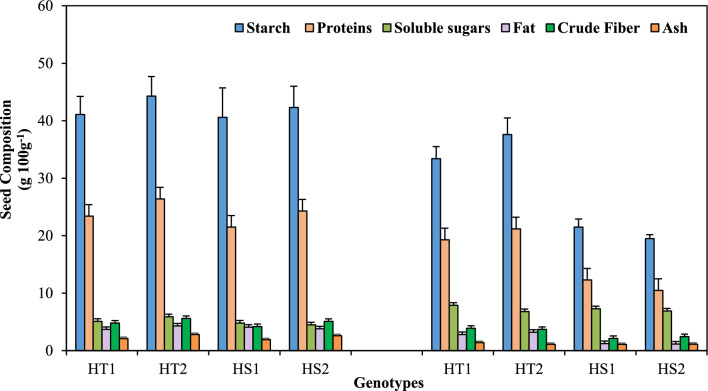
Seed composition in heat-tolerant (HT1: ICCV07110; HT2: ICCV92944) and heat-sensitive (HS1: ICC14183; HS2: ICC5912) chickpea genotypes grown under control (25/15 °C) and heat stress (32/20 °C) environment. LSD: (*P* < 0.05) (genotypes x treatment interaction): Starch: 3.65; Proteins: 2.14; Soluble sugars: 0.87; Fat: 0.24; Crude fiber: 0.38; Ash: 0.32.

The various seed components (Fig. 6, Tables 1, 2) generally decreased with heat stress exposure in both genotypes compared with their respective controls. Heat stress decreased the starch content by 47–54% in the HS genotypes and 15 -18% in the HT genotypes. Seed protein content decreased by 42.8–56.7% in the HS genotypes and 17.5–19.7% in the HT genotypes. The fat content decreased by about 69.7% in the HS genotypes and 24.3–25. 8% in HT genotypes. Crude fiber and ash contents also decreased more in the HS genotypes than in the HT genotypes. However, there was a significant increase in the soluble sugar content in the HT and HS genotypes.

### Seed storage proteins

**Figure 7:**
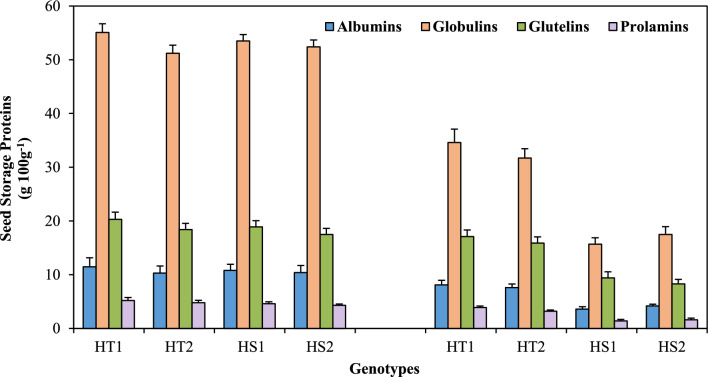
Seed storage proteins in heat-tolerant (HT1: ICCV07110; HT2: ICCV92944) and heat-sensitive (HS1: ICC14183; HS2: ICC5912) chickpea genotypes grown under control (25/15 °C) and heat stress (32/20 °C) environment. LSD: (*P* < 0.05) (genotypes x treatment interaction): Albumins: 1.76, Globulins: 6.12; Glutelins: 2.13; Prolamins: 0.78.

Heat stress severely reduced various seed storage proteins (Fig. 7, Tables 1, 2), more so in the HS genotypes than in the HT genotypes, compared to their respective controls. Heat stress decreased albumins by 59.6–66.7% in the HS genotypes and 26.2–29.5% in the HT genotypes. The globulins decreased by 66.6–70.6% in the HS genotypes and 37.2–38.0% in the HT genotypes. Glutelins showed a 50.2–52.5% reduction in the HS genotypes and a 13.5–15.7% reduction in the HT genotypes. At the same time, prolamins exhibited a 62.57–69.5% reduction in the HS genotypes and a 25–33% reduction in the HT genotypes.

### Seed carbohydrate metabolism

**Figure 8:**
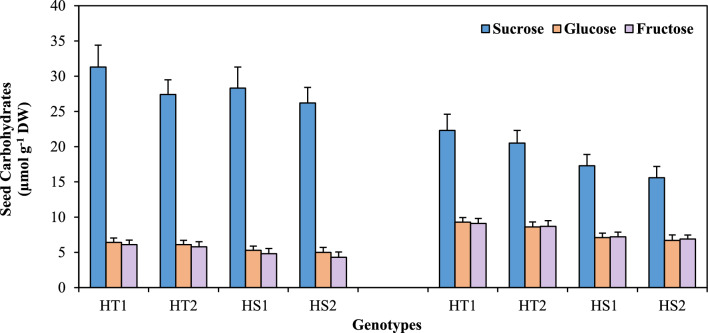
Seed carbohydrates in heat-tolerant (HT1: ICCV07110; HT2: ICCV92944) and heat-sensitive (HS1: ICC14183; HS2: ICC5912) chickpea genotypes grown under control (25/15 °C) and heat stress (32/20 °C) environment. LSD: (*P* < 0.05) (genotypes x treatment interaction): Sucrose: 1.13, Glucose: 0.41; Fructose: 0.39.

The sucrose concentration (Fig. 8, Tables 1, 2) in the seeds of heat-stressed plants decreased more in the HS genotypes (53–55%) than in the HT genotypes (25–28.7%). In contrast, heat stress markedly increased glucose and fructose concentrations by 41–45% and 49–50% in the HT genotypes and 33–35% and 50–60% in the HS genotypes, respectively, relative to their corresponding controls.

Heat stress decreased activities of enzymes-sucrose synthase, soluble starch synthase, and acid invertases (Tables 1, 2, 4), more so in the HS genotypes (46.4–46.8%, 40–45%, and 42.5–45.4%, respectively) than in the HT genotypes (17.6–18.2%, 17–22%, and 19.3–22.6%, respectively) in relation to their respective controls.

**Table 4:** Enzymes related to carbohydrate metabolism in seeds of heat-tolerant (HT1: ICCV07110; HT2: ICCV92944) and heat-sensitive (HS1: ICC14183; HS2: ICC5912) chickpea genotypes grown in control (25/15 °C) and heat stress (32/20 °C) environment.

Enzymes (nmol min^–1^ mg^–1^ protein^–1^)	Control				Stressed			
	HT1	HT2	HS1	HS2	HT1	HT2	HS1	HS2
Sucrose synthase (SS)	32.4 ± 2.45a	28.4 ± 2.12a	26.7 ± 1.89b	23.5 ± 1.54bc	26.5 ± 1.35b	23.4 ± 1.18bc	14.3 ± 1.12c	12.4 ± 1.13c
Soluble starch synthase (Ssy)	1324.4 ± 28.6a	1231.3 ± 31.4b	1134.5 ± 22.5c	1054.5 ± 25.7d	1034.5 ± 14.5e	1023.4 ± 16.7e	675.6 ± 11.4f.	576.8 ± 9.4f.
Acid invertases (AI)	1132.4 ± 36.6a	1054.5 ± 45.3b	965.6 ± 31.3c	941.3 ± 33.6c	913.4 ± 25.7d	815.6 ± 21.4d	554.5 ± 25.9e	513.5 ± 34.5e

**LSD (P** <  < 0.05) for genotypes x treatment interaction: SS (3.2), Ssy (33.4), AI (47.5), Values represent mean ± S.E. Different lower-case letters for each trait indicate significant variations (*P* < 0.05).

### Mineral nutrients

The heat stress treatments had a significant impact on the seed Ca, P, and Fe concentrations of both HS and HT genotypes compared to the controls, with decreases of 47–54% for Ca, 33–37% for P, and 59% for Fe in HS genotypes, and 21–25% for Ca, 21–25% for P, and 31–35% for Fe in HT genotypes (Table 1, 2, and 5).

**Table 5:** Seed minerals in heat-tolerant (HT1: ICCV07110; HT2: ICCV92944) and heat-sensitive (HS1: ICC14183; HS2: ICC5912) chickpea genotypes grown in control (25/15 °C) and heat stress (32/20 °C) environments.

Minerals (mg 100 g^–1^)	Control				Stressed			
	HT1	HT2	HS1	HS2	HT1	HT2	HS1	HS2
Ca	45.6 ± 3.2a	41.6 ± 2.8a	39.4 ± 2.6bc	36.7 ± 2.1c	34.2 ± 2.8c	32.4 ± 2.1c	17.9 ± 1.9d	19.4 ± 2.1d
P	156.3 ± 6.4a	124.5 ± 5.8c	141.2 ± 8.5b	138.4 ± 6.7bc	113.4 ± 9.4 cd	107.4 ± 6.4 cd	89.3 ± 7.6d	92.3 ± 6.9d
Fe	3.16 ± 0.25a	2.87 ± 0.21a	2.95 ± 0.22a	2.84 ± 0.31bc	2.18 ± 0.34c	1.87 ± 0.15c	1.21 ± 0.15d	1.14 ± 0.13d

**LSD (P < ** < 0.05) for genotypes x treatment interaction: Ca (4.6), P (9.8), Fe (0.41), Values represent mean ± S.E. Different lower case letters for each trait indicate significant variations (P < 0.05).

## Discussion

Heat stress during the seed-filling stage had a significant negative impact on chickpea seed yield and quality, particularly in the HS genotypes compared to the HT genotypes. Reduced seed numbers under heat stress have been attributed to a decrease in pod numbers caused by fertilization failure, as previously reported for chickpea^25,59^. Heat stress also led to a substantial decline in single-seed weight owing to disruptions in the accumulation of various seed components, resulting in decreased seed weight per plant and harvest index. These findings align with those of previous studies highlighting the detrimental effects of high temperatures on yield-related traits in chickpea^59,60^ and other crops, such as wheat^61^ and maize^62^. Additionally, heat stress adversely affected seed nutritional components, including carbohydrates, proteins, and fats, leading to poor seed quality^3,5,13,30,36^.

Heat stress impairs vegetative growth by damaging leaf tissues, disrupting of various functions and eventually inhibiting seed development^10^. Membrane damage in leaves is a reliable indicator of thermotolerance in legumes^63^. High temperatures disrupt membrane organization or generate reactive oxygen species (ROS)^64^ that target the cell membranes. Heat stress resulted in reduced leaf chlorophyll content and necrosis, which is consistent with observations in bent grass^65^. A reduction in chlorophyll content can occur because of the inhibition of biosynthesis and/or photooxidation. Tissue death caused by heat stress is likely a direct consequence, as reported in other plant species, such as the common bean^66^. Moreover, heat stress caused a significant decrease in chlorophyll fluorescence, indicating impaired photosynthetic efficiency, similar to the findings in heat-stressed cotton^67^ and lentil^7^. Heat stress severely disrupted the carbon fixation ability, as measured by RuBisCo activity, in chickpea, which was associated with reduced stomatal conductance or inhibited RuBisCo activity^68^. Heat stress also decreased sucrose synthase activity, limiting carbon assimilation, and subsequently reducing leaf sucrose content^8,68^. Another study reported similar inhibition of photosynthesis, RuBisCo activity, and sucrose synthase activity in heat-stressed lentil plants^69^.

Leaf water status, indicated by relative leaf water content (RLWC), was significantly decreased in heat-stressed chickpea plants, suggesting the impact of intensified water stress on the aforementioned leaf traits under high temperatures^70^. A previous study also reported a reduction in RLWC in chickpea plants grown in a high-temperature environment. In our study, heat stress increased stomatal conductance in the HT genotypes but decreased it in the HS genotypes, which explains the higher RLWC values in the HT genotypes. Water loss from heat-stressed chickpea leaves emerged as a crucial factor affecting cellular metabolism and differentiating heat sensitivity among the contrasting genotypes. The HT chickpea genotypes exhibited higher RLWC and stomatal conductance under heat stress than the HS genotypes, resulting in greater photosynthetic activity. Previous studies have highlighted the effect of leaf water status on heat tolerance in mung bean^71^ and alfalfa plants^9^.

Heat stress significantly affects seed filling in chickpea, leading to a marked reduction in seed-filling rate and duration, thereby inhibiting the accumulation of various constituents such as starch, proteins, and fats. The decrease in these seed components could result from disrupted import of precursors from leaves, decreased biosynthesis in seeds, or both. Sucrose import into seeds can be significantly inhibited in heat-stressed plants because of reduced availability in leaves and decreased expression of sucrose transporters^72^. We observed marked inhibition of sucrose synthase and soluble starch synthase activities in chickpea seeds, leading to reduced carbohydrate content and smaller seeds. In wheat, heat stress decreases the activities of sucrose synthase, soluble starch synthase, glucokinase, and ADP glucose pyrophosphorylase, resulting in poor starch and fat accumulation^73^, which is similar to the findings in maize^34,74^. Similarly, heat stress decreases the activities of sucrose synthase and starch branching enzymes in aromatic rice grains, impairing starch accumulation^75^.

The decrease in proteins, including storage proteins, could be attributed to insufficient precursors and/or inhibited biosynthetic enzyme activities^76^, which is consistent with previous studies on maize^77^, lentil^7^, and soybean^36^ grown in high-temperature environments. In contrast, maize seeds exhibit increased protein levels under heat stress because of the increased activity of associated enzymes^34^. Downregulation of seed storage protein-encoding genes under heat stress could be another possible reason for the decline in storage protein in chickpea, as evidenced in soybean^36^. The differing observations in this regard may be attributed to variations in heat stress treatments across the studies.

Fat accumulation decreased significantly in heat-stressed chickpea seeds, possibly because of a decline in acetyl-CoA content resulting from inhibited photosynthetic activity, as reported in common bean^77^ and canola^78^. Further research is required to understand the mechanisms that affect protein and fat synthesis in chickpea seeds at high temperatures.

Heat stress inhibits the accumulation of minerals (Ca, Fe, and P) in chickpea seeds, possibly because of impaired translocation from leaves^79^, which disrupts transport mechanisms in seeds and the mobilization of various molecules and ions. Further investigation is necessary to understand the transport mechanisms of these minerals in chickpea seeds developing under high-temperature environments.

Observations of contrasting chickpea genotypes under heat stress revealed that the heat-tolerant (HT) genotypes exhibited better leaf maintenance, less membrane damage to their photosynthetic activity, higher water retention and greater stomatal conductance. Consequently, heat stress affected leaf sucrose generation less in the HT genotypes than in the HS genotypes, resulting in more sucrose being available for export to seeds. Furthermore, the HT genotypes demonstrated more stable enzymes for sucrose and starch synthesis than the HS genotypes, leading to increased starch and sucrose contents. Similar implications can be speculated for other storage components such as proteins and fats, which require further investigation. Nonetheless, our observations of contrasting chickpea genotypes have provided valuable insights into the target sites of heat stress in the leaves and seeds, which can be leveraged in the development of heat-tolerant chickpea cultivars.

In conclusion, heat stress during the chickpea seed-filling stage negatively affected seed yield and quality, particularly in heat-sensitive genotypes. Heat stress reduced seed numbers by decreasing pod numbers because of fertilization failure. It also led to a smaller seed size and decreased the accumulation of carbohydrates, proteins, and fats. Heat stress disrupted leaf chlorophyll content, photosynthetic efficiency, and carbon fixation, causing a decrease in leaf water content and impaired seed filling, resulting in reduced starch, protein, and fat levels. Heat stress also inhibited mineral accumulation in the seeds. The contrasting genotypes revealed the importance of improved leaf maintenance and water retention in heat-tolerant genotypes. These findings highlight the need for heat-tolerant cultivar development and further research to understand its underlying mechanisms.

## Data Availability

The data related to the findings of this study are available within the article.
